# Inequality in child undernutrition among urban population in India: a decomposition analysis

**DOI:** 10.1186/s12889-020-09864-2

**Published:** 2020-12-03

**Authors:** S. K. Singh, Shobhit Srivastava, Shekhar Chauhan

**Affiliations:** 1 Department of Mathematical Demography and Statistics, International Institute for Population Sciences, Mumbai, Maharashtra 400088 India; 2 Department of Population Policies and Programmes, International Institute for Population Sciences, Mumbai, Maharashtra 400088 India

**Keywords:** Underweight, Stunting, Wasting, Economic inequality, India

## Abstract

**Background:**

With increasing urbanization in India, child growth among urban poor has emerged as a paramount public health concern amidst the continuously growing slum population and deteriorating quality of life. This study analyses child undernutrition among urban poor and non-poor and decomposes the contribution of various factors influencing socio-economic inequality. This paper uses data from two recent rounds of National Family Health Survey (NFHS-3&4) conducted during 2005–06 and 2015–16.

**Methods:**

The concentration index (CI) and the concentration curve (CC) measure socio-economic inequality in child growth in terms of stunting, wasting, and underweight. Wagstaff decomposition further analyses key contributors in CI by segregating significant covariates into five groups-mother’s factor, health-seeking factors, environmental factors, child factors, and socio-economic factors.

**Results:**

The prevalence of child undernutrition was more pronounced among children from poor socio-economic strata. The concentration index decreased for stunting (− 0.186 to − 0.156), underweight (− 0.213 to − 0.162) and wasting (− 0.116 to − 0.045) from 2005 to 06 to 2015–16 respectively. The steepness in growth was more among urban poor than among urban non-poor in every age interval. Maternal education contributed about 19%, 29%, and 33% to the inequality in stunting, underweight and wasting, respectively during 2005–06. During 2005–06 as well as 2015–16, maternal factors (specifically mother’s education) were the highest contributory factors in explaining rich-poor inequality in stunting as well as underweight. More than 85% of the economic inequality in stunting, underweight, and wasting among urban children were explained by maternal factors, environmental factors, and health-seeking factors.

**Conclusion:**

All the nutrition-specific and nutrition-sensitive interventions in urban areas should be prioritized, focusing on urban poor, who are often clustered in low-income slums. Rich-poor inequality in child growth calls out for integration and convergence of nutrition interventions with policy interventions aimed at poverty reduction. There is also a need to expand the scope of the Integrated Child Development Services (ICDS) program to provide mass education regarding nutrition and health by making provisions of home visits of workers primarily focusing on pregnant and lactating mothers.

**Supplementary Information:**

The online version contains supplementary material available at 10.1186/s12889-020-09864-2.

## Background

The rapid increase in the urban population due to urbanization in the last one decade has contributed to the urban poverty in India [47]. It is manifested in the form of inadequate provision of housing and shelter, water, sanitation, health, education, social security, livelihoods, and food security along with the special needs of vulnerable groups like women, and children [13]. The health of urban children as measured with malnutrition in the existing context of urbanization, has become an important issue in India [26]. Malnutrition among children under age five is a paramount public health concern in many developing countries, and India is no exception [33].

Child under-nutrition is expressed in different forms, including stunting (− > 2 SD length/weight for age), wasting (− > 2 SD weight for length/height), and underweight (− > 2 SD weight for age) [43]. In developing countries, an estimated 32% of children under five were stunted, 3.5% were severely wasted, and 20.2% were underweight [4]. The rate of stunting in India has declined from 48% to 38.4%, wasting has increased slightly from 19.8% to 21%, and underweight has decreased from 42.5% to 35.8% between the two time-periods of NFHS-3 and NFHS-4 [17]. Malnutrition in India is clustered in central, northern, and eastern regions [46]. In a study conducted in Indian settings, it was found that the spatial clustering of malnutrition was visible in those geographical pockets where poverty was high, women’s education was low, and BMI level among women was below average [22].

Malnutrition is primarily caused due to the immediate causes (inadequate dietary intake, lack of care and onset of disease), and the underlying causes (an unhealthy environment and insufficient education) [9]. Studies in countries other than India had also raised the issue of child growth significantly. In a study, it was found that there was a substantial poor/rich gap in the nutritional status in the urban residence of Kenya [11]. Moreover, the same study argued that poor nutritional status in urban areas is attributed to poor environment and housing, poor access to drinking water, food, and health services. One of the studies found that the children belonging to poor households were undernourished not only because of poverty, but also due to limited use of maternal health care services and poor care resulting from the lower educational status of parents and poor health of mothers [5].

Despite high levels of stunting, wasting, and underweight among under-five children in India, studies related to measuring growth faltering as measured with z score are very limited [14]. Studies have found that growth in weight appeared to start right from birth, and there appeared a fall in WAZ score for the first 3 months, and after that, the rate of fall appeared to slow down [25]. The study was done to compare NFHS-1 and NFHS-3 data to provide a comparison for all India level growth among children aged 0–3 years [25]. Growth in infancy was associated with poorer educational outcomes in later childhood. However, these outcomes, when adjusted for confounding factors, suggested that childhood educational attainment was more strongly influenced by other socio-demographic factors, especially maternal education and the quality of the home environment [15, 42]. Similarly, it was found that early childhood stunting is associated with deficits in cognition and educational achievement in late adolescence [42].

India ranks 102 in the Global Hunger Index [12], shows the severity of under-nutrition existence in the country. Moreover, other studies too carved out the fact that the poverty nutrition trap does exist in India [19]. There exist lack of literature focusing on examining the socio-economic gap for malnutrition among children in urban India. Therefore, this paper aims to measure undernutrition among urban poor and non-poor children below age five in India. It also analyses the changes in socio-economic inequality in undernutrition among children in urban India from 2005 to 06 to 2015–16.

## Methods

### Data

The data used in this paper have been taken from two rounds of National Family Health Survey namely NFHS-3 (2005–06) and NFHS-4 (2015–16). The primary objective of the NFHS was to provide essential data on health and family welfare, as well as data on emerging issues including the nutritional status of children under age five at national, state, and district levels. Therefore, data from NFHS-4 (2015–16) was considered as a benchmark to examine the progress made by the country in various health indicators over time. Decisions about the overall sample size required for National Family Health Survey (2005–06 and 2015–16) were guided by several considerations, paramount among which was the need to produce indicators at the national levels, as well as separate estimates for urban and rural areas. The data is a nationally representative data which provide essential data on health and family welfare, as well as data on emerging issues in these areas. Additionally, National Family Health Survey is a part of Demographic and Health Surveys (DHS) which are nationally-representative household surveys that provide data for a wide range of monitoring and impact evaluation indicators in the areas of population, health, and nutrition. The findings of this study are based on a total of 15,241 urban children under 5 years in NFHS-3 and 53,483 urban children under five years in NFHS-4. The information related to child’s anthropometric measures covered measurements of height, weight and hemoglobin levels for children; Details about the NFHS-4 designs, tools, and protocols are presented in the national report of NFHS-4 [17], and all relevant information is available in the public domain on http://rchiips.org/ NFHS.

### Categorization of variables

#### Outcome variable

The three anthropometric indicators were used for children under five years: stunting (height for age < − 2 standard deviation (SD)), underweight (weight for age < − 2 SD), and wasting (height for age < − 2 SD). The measure used was recoded as per WHO standards [45].

#### Predictor variable

The study divided the predictors into five groups. 1) Maternal Factors 2) Socio-economic factors 3) Child-related factors 4) Environmental factors and 5) Health seeking factors. Maternal factors include age (15–24, 25–24 and 35+ years), Education (Illiterate and literate), age at first birth (< 18, 18–30, and > 30 years), Parity (1, 2, 3, 4+) and mother’s anaemia status (no and yes). Socio-economic factors include caste (SC/ST and non-SC/ST), religion (Hindu, Muslim, Christian, and Others) and Wealth status (Poorest, Poorer, Middle, Richer, and Richest). Child factors consist age (0–23, 24–47 months and 47–59 months), sex of the child (male and female), anemia status (no and yes), diarrhea, birth weight (normal and low birth weight (<2500grms), and colostrum feeding (yes and no). The environmental factors include the source of drinking water (protected and not protected), open defecation (opened and improved), stool disposal (yes and no), and geographical regions (North, Central, East, North-east, West, and South). Health seeking factors included the use of Skill Birth Attendants, Place of delivery (Home or any institution i.e., private/public hospital), full immunization includes children who received BCG (for tuberculosis), measles, and three doses each of DPT (diphtheria, whooping cough, and tetanus), and Polio. Full ANC is defined as women who had four or more ANC visits, had at least two tetanus injection, and consumed 100 IFA tablets/syrup for the most recent birth. Further, Colostrum feeding, defined as a child received breastfeeding within 1 h after delivery.

The variable of wealth status was created using the information given in the survey. Households were given scores based on the number and kinds of consumer goods they own, ranging from a television to a bicycle or car, and housing characteristics such as source of drinking water, toilet facilities, and flooring materials. These scores are derived using principal component analysis. National wealth quintiles are compiled by assigning the household score to each usual (de jure) household member, ranking each person in the household population by their score, and then dividing the distribution into five equal categories, each with 20% of the population. Further, wealth status of the household was recoded as poor and non-poor. To depict the poor and non-poor differentials, we clubbed first two wealth quintiles as poor (poorest and poorer) and next three quintiles as non-poor (middle, richer and richest).

### Statistical analysis

#### Concentration index (CI)

The concentration curve is obtained by plotting the cumulative proportion of outcome variables (either stunting, underweight, or wasting) on y-axis against the increasing percentage of the population ranked by the socio-economic indicator (wealth index) on x-axis. The curves shows that whether the socio- economic status related inequality in the outcome variable (on x-axis) prevails or not. If the curve is above the line of equality (45 degree line) that means the index value is negative hence it shows that the outcome variable is disproportionally concentrated among the poor and vice-versa. Income-related inequality in stunting, underweight, and wasting was measured by the concentration index (CI) and the concentration curve (CC), using the wealth score as the socio-economic indicator and binary outcome as stunting, underweight, and wasting. The concentration index is defined as twice the area between the concentration curve and the line of equality. Concentration index measures the inequality of one variable (say stunting, underweight or wasting) over the distribution of another variable (wealth index). The index ranges from − 1 to + 1, where the index value of 0 (zero) shows no socio-economic inequality. However, positive value of index shows pro-rich inequality and vice-versa. Additional on the either scales higher the value, higher the extent of socio-economic inequality. The study used Wagstaff decomposition analysis to decompose the concentration index. Wagstaff’s decomposition demonstrated that the concentration index could be decomposed into the contributions of each factor to the income-related inequalities [41]. For any linear regression model on health outcome (y) (say stunting, underweight or wasting), such as.

1 $$ y=\alpha +{\sum}_k{\beta}_k{x}_k+\varepsilon $$

The concentration index for y, C, can be written as follows,

2 $$ C={\sum}_k\left({\beta}_k{\overline{x}}_k/\mu \right){C}_k+G{C}_{\varepsilon }/\mu $$

Where *μ* is the mean of y, ${\overline{x}}_k$ is the mean of *x*_*k*_, *C*_*k*_ is the concentration index for *x*_*k*_ (defined analogously to C), and *GC*_*ε*_ is the generalized concentration index for the error term (*ε*). Equation (2) shows that C is equal to a weighted sum of the concentration indices of the k regressors, where the weight for *x*_*k*_ is the elasticity of y with respect to *x*_*k*_ $\left({\eta}_k={\beta}_k\frac{{\overline{x}}_k}{\mu}\right)$. The residual component captured by the last term reflects the socio-economic status related inequality in health that is not explained by systematic variation in the regressors by income, which should approach zero for a well-specified model. Each contribution is the product of elasticity with the degree of economic inequality. Moreover, the percentage contribution is obtained by dividing each absolute contribution by total absolute contribution multiplied by 100 to obtain the estimates.

## Results

Table 1 shows the distribution of children under age five by selected background characteristics among urban poor and non-poor in India. It was found that significant health problems like anaemia and diarrhoea among under-five children were more prevalent among those from urban poor as compared to those from urban non-poor. On contrary, health-seeking behaviour was better practised by urban residents as compared to non-poor population.

**Table 1:** Percentage distribution of children under age five years by selected background characteristics among urban poor and non-poor in India, 2005–06 and 2015–16.

Variables	2005–06				2015–16			
	Non-poor		Poor		Non-poor		Poor	
	N	%	N	%	N	%	N	%
**Age (years)**								
15–24	4502	32.9	607	39.2	12,235	27.0	2843	34.6
25–34	8099	59.2	756	48.8	28,657	63.3	4443	54.1
35+	1092	8.0	185	12.0	4374	9.7	931	11.3
**Education**								
No	2711	19.8	1023	66.1	5357	11.8	3747	45.6
Yes	10,982	80.2	525	33.9	39,909	88.2	4470	54.4
**Age at First Birth (years)**								
Less than 18	2210	16.1	626	40.44	3503	7.7	1561	19.0
18–30	11,002	80.4	910	58.8	40,202	88.8	6520	79.4
More than 30	481	3.5	12	0.78	1561	3.5	136	1.7
**Parity**								
1	3577	26.1	223	14.4	14,131	31.2	1506	18.3
2	5334	39.0	344	22.2	18,883	41.7	2515	30.6
3	2423	17.7	300	19.4	7620	16.8	1901	23.1
4+	2359	17.2	681	44.0	4632	10.2	2295	27.9
**Anemia**								
No	7045	51.5	592	38.2	22,264	49.2	3312	40.3
Yes	6648	48.6	956	61.8	23,002	50.8	4905	59.7
**Socio-economic factors**								
**Caste**								
SC/ST	3406	24.9	565	36.5	12,103	26.7	3269	39.8
Non-SC/ST	10,287	75.1	983	63.5	33,163	73.3	4948	60.2
**Religion**								
Hindu	9143	66.8	1013	65.4	29,762	65.8	5508	67.0
Muslim	2714	19.8	413	26.7	10,077	22.3	2024	24.6
Others	1836	13.4	122	7.9	5427	12.0	685	8.3
**Child factors**								
**Age (months)**								
0–23	5115	37.4	576	37.2	17,199	38.0	3071	37.4
24–47	5741	41.9	639	41.3	18,777	41.5	3399	41.4
47–59	2837	20.7	333	21.5	9290	20.5	1747	21.3
**Sex**								
Male	7223	52.8	748	48.3	23,666	52.3	4221	51.4
Female	6470	47.3	800	51.7	21,600	47.7	3996	48.6
**Anemia**								
No	7208	52.6	604	39.0	23,501	51.9	3719	45.3
Yes	6485	47.4	944	61.0	21,765	48.1	4498	54.7
**Diarrhea**								
No	12,409	90.6	1396	90.2	41,256	91.1	7411	90.2
Yes	1284	9.4	152	9.8	4010	8.9	806	9.8
**Birth weight**								
Normal	12,003	87.7	1462	94.4	38,744	85.6	7086	86.2
LBW	1690	12.3	86	5.6	6522	14.4	1131	13.8
**Colostrum feeding**^**a**^								
No	8925	65.2	1136	73.4	30,234	66.8	5634	68.6
Yes	4768	34.8	412	26.6	15,032	33.2	2583	31.4
**Environmental factors**								
**Source of drinking water**								
Not Protected	1097	8.0	211	13.6	3889	8.6	965	11.7
Protected	11,840	86.5	1259	81.3	39,039	86.2	6842	83.3
Others	756	5.5	78	5.0	2338	5.2	410	5.0
**Defecation**								
Open defecation	1089	8.0	940	60.7	2725	6.0	4309	52.4
Improved	11,790	86.1	511	33.0	40,065	88.5	3456	42.1
Others	814	5.9	97	6.3	2476	5.5	452	5.5
**Stool Disposal**								
Yes	11,382	83.1	824	53.2	36,300	80.2	4235	51.5
No	2308	16.9	724	46.8	8955	19.8	3982	48.5
**Regions of India**								
North	3029	22.1	315	20.4	10,054	22.2	910	11.1
Central	2032	14.8	166	10.7	12,640	27.9	2632	32.0
East	563	4.1	25	1.6	4634	10.2	2324	28.3
North East	3023	22.1	361	23.3	5675	12.5	1165	14.2
West	2823	20.6	496	32.0	4745	10.5	547	6.7
South	2223	16.2	185	12.0	7518	16.6	639	7.8
**Health seeking factors**								
**Skilled birth attendant**^**a**^								
No	2987	21.8	986	63.7	3950	8.7	2148	26.1
Yes	10,706	78.2	562	36.3	41,316	91.3	6069	73.9
**Place of delivery**^**a**^								
Home	3757	27.4	1084	70.0	4629	10.2	2428	29.6
Institution	9936	72.6	464	30.0	40,637	89.8	5789	70.5
**Full Immunization**								
No	5643	41.2	1088	70.3	15,158	33.5	3713	45.2
Yes	8050	58.8	460	29.7	30,108	66.5	4504	54.8
**Full ANC**^**a**^								
No	11,303	82.6	1494	96.5	35,184	77.7	7501	91.3
Yes	2378	17.4	54	3.5	10,082	22.3	716	8.7

*SC/ST* Scheduled Caste/Scheduled Tribe; *ANC* Ante-Natal Care

^a^*estimates only for last birth; N: Sample; % Percentage*

**Fig. 1:**
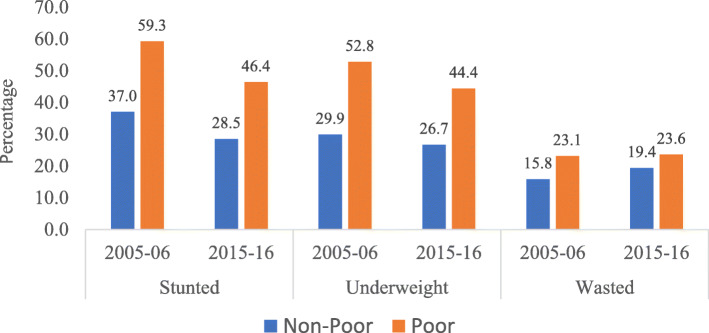
Poor and non-poor differentials for malnutrition among children under five years in urban India, 2005–06 and 2015–16

Figure 1 portrays the prevalence of child growth as measured through stunting, wasting, and underweight among urban poor and non-poor in India, during 2005–06 and 2015–16. It is evident from the results that three key indicators of growth among children under age five, namely stunting, underweight, and wasting were higher among urban poor children than among urban non-poor children for both the time-periods. Further, results show that the prevalence of stunting and underweight has declined among urban poor as well as non-poor children during the last one decade, i.e., from 2005 to 06 to 2015–16. However, the prevalence of wasting has increased, from 2005 to 06 to 2015–16, among urban poor as well as urban non-poor children, which may be primarily due to seasonal variation in the month of surveys. By and large, a similar result has been found in case of growth for poor and non-poor children in urban India during 2005–06 and 2015–16. The result found that the average growth is higher among urban poor than in urban non-poor for each classification of child anthropometry measures (Figure-S1; Supplementary file). For standard error, please refer supplementary *Table S**1**and S**2*.

Table 2 presents the average growth among children as measured through z scores for HAZ, WHZ, and WAZ by segregating them into different age groups. Mean WAZ, HAZ and WHZ scores for children under 5 months were (− 0.95, − 1.54, − 1.11), and (− 0.36,-1.05, − 1.00) in 2005–06; and (− 0.86, − 1.49, − 1.09), and (− 0.33, − 1.18, − 1.25) in 2015–16 for urban poor and non-poor respectively. Significant differences in z score in poor as well as non-poor children were visible from the first 5 months itself. Of the total growth differential as measured with z score for height-for-age (HAZ) at 59 months among urban poor and non-poor in 2005–06 (− 2.51 and − 1.59), 38% (− 0.95) and 22% (− 0.36) of growth took place at first 5 months after birth; whereas, in 2015–16, the distribution increased to 41% (− 0.86) and 25% (− 0.33) respectively. Similarly, of the average growth in weight-for-age (WAZ) at 59 months among urban poor and non-poor in 2005–06 (− 2.18 and − 1.54), 71% (− 1.54) and 68% (− 1.05) of growth took place at first 5 months after birth; whereas, in 2015–16, the distribution was 41% (− 0.86) and 25% (− 0.33) respectively. For weight-for-height (WHZ) at 59 months among urban poor and non-poor in 2005–06 (− 0.99 and − 0.88), 112% (− 1.11) and 113% (− 1.00) of growth took place at first 5 months after birth; whereas in 2015–16, the distribution was 99% (− 1.09) and 131% (− 1.25) respectively. Growth for HAZ among children was found to be highest at age 21–25 months among urban poor, and at age 46–50 months among urban non-poor during 2005–06, whereas, growth in stunting was highest at age 21–25 months among urban poor and non-poor during 2015–16. It leads to the premise that the children were more susceptible to be stunted during the age of 21–25 months. Z score for WAZ among children was found to be highest at age 31–35 months among urban poor and at age 56–59 months among urban non-poor during 2005–06, whereas, z score for underweight was highest at age 21–25 months among urban poor and at age 56–59 months among urban non-poor during 2015–16. It means that urban non-poor children aged 56–59 months were more prone to be underweight. The z scores for WHZ among children was found to be highest at age 6–10 months among urban poor and at age 0–5 months among urban non-poor during 2005–06, whereas, z score for wasting was highest at age 6–10 months among urban poor and at age 0–5 months among urban non-poor during 2015–16. It means that children in their early months were more prone to be wasted.

**Table 2:** Mean height for age z (HAZ), weight for age z (WAZ), and weight for height z (WHZ) scores among children under five years in urban India, 2005–06 and 2015–16

Age (in Months)	2005–06						2015–16					
	Urban Poor			Urban Non-poor			Urban Poor			Urban Non-poor		
	n	Mean	SD	n	Mean	SD	n	Mean	SD	n	Mean	SD
**0–5 months**												
HAZ	100	−0.95	1.78	773	− 0.36	1.90	521	−0.86	1.91	2808	− 0.33	1.88
vWAZ	100	−1.54	1.63	773	−1.05	1.30	521	−1.49	1.32	2808	−1.18	1.31
WHZ	100	− 1.11	1.75	773	−1.00	1.91	521	−1.09	1.79	2808	−1.25	1.84
**5–10 months**												
HAZ	127	−1.41	1.97	1153	−0.61	1.68	712	− 0.73	2.07	3784	−0.33	1.85
WAZ	127	−2.06	1.43	1153	−1.08	1.28	712	−1.56	1.38	3784	−1.06	1.27
WHZ	127	− 1.44	1.62	1153	−0.85	1.57	712	−1.34	1.57	3784	− 1.02	1.63
**10–15 months**												
HAZ	112	− 1.82	1.53	1105	−1.14	1.58	612	−1.51	1.89	3600	− 0.88	1.90
WAZ	112	−1.91	1.26	1105	− 1.19	1.21	612	−1.71	1.30	3600	−1.10	1.26
WHZ	112	−1.32	1.43	1105	−0.82	1.43	612	−1.22	1.49	3600	−0.84	1.52
**15–20 months**												
HAZ	157	−2.24	1.76	1172	−1.62	1.60	702	−1.74	1.83	3977	−1.27	1.72
WAZ	157	− 2.04	1.30	1172	−1.27	1.24	702	−1.75	1.22	3977	−1.18	1.22
WHZ	157	−1.30	1.47	1172	−0.68	1.32	702	−1.24	1.30	3977	−0.78	1.41
**20–25 months**												
HAZ	100	−2.76	1.52	1133	−1.76	1.56	656	−2.15	1.63	3771	−1.42	1.73
WAZ	100	−2.19	1.39	1133	−1.47	1.17	656	−2.01	1.16	3771	−1.28	1.29
WHZ	100	−1.10	1.49	1133	−0.81	1.22	656	−1.27	1.23	3771	−0.79	1.41
**25–30 months**												
HAZ	138	−2.33	1.61	1227	−1.61	1.54	667	−1.94	1.68	3819	−1.07	1.74
WAZ	138	−2.05	1.10	1227	−1.38	1.18	667	−1.88	1.17	3819	−1.23	1.21
WHZ	138	−1.10	1.10	1227	−0.73	1.21	667	−1.17	1.29	3819	−0.94	1.28
**30–35 months**												
HAZ	134	−2.61	1.81	1196	−1.72	1.61	697	−1.92	1.48	3877	−1.38	1.52
WAZ	134	−2.25	1.22	1196	−1.49	1.23	697	−1.82	1.14	3877	−1.34	1.19
WHZ	134	−1.10	1.20	1196	−0.77	1.29	697	−1.07	1.21	3877	−0.85	1.29
**35–40 months**												
HAZ	133	−2.52	1.61	1227	−1.66	1.64	710	−1.97	1.49	3900	−1.28	1.53
WAZ	133	−2.13	1.00	1227	−1.53	1.27	710	−1.91	1.08	3900	−1.37	1.20
WHZ	133	−0.98	1.02	1227	−0.86	1.24	739	−1.14	1.33	3900	−0.95	1.34
**40–45 months**												
HAZ	134	−2.46	1.56	1219	−1.70	1.50	739	−1.91	1.54	4041	−1.23	1.46
WAZ	134	−2.01	1.35	1219	−1.50	1.11	739	−1.89	1.16	4041	−1.28	1.21
WHZ	134	−0.83	1.47	1219	−0.75	1.18	710	−1.13	1.28	4041	−0.85	1.32
**45–50 months**												
HAZ	131	−2.48	1.68	1098	−1.85	1.63	749	−1.93	1.42	3976	−1.31	1.45
WAZ	131	−2.10	1.47	1098	−1.69	1.20	749	−1.98	1.06	3976	−1.39	1.14
WHZ	131	−0.90	1.50	1098	−0.87	1.25	749	−1.24	1.19	3976	−0.92	1.33
**50–55 months**												
HAZ	145	−2.62	1.20	1238	−1.46	1.39	718	−1.91	1.34	3883	−1.28	1.35
WAZ	145	−2.22	1.06	1238	−1.39	1.17	718	−1.83	1.09	3883	−1.35	1.21
WHZ	145	−0.95	1.26	1238	−0.78	1.27	718	−1.03	1.18	3883	−0.89	1.39
**55–60 months**												
HAZ	137	−2.51	1.29	1152	−1.59	1.36	734	−2.08	1.20	3830	−1.34	1.26
WAZ	137	−2.18	1.02	1152	−1.54	1.16	734	−1.98	0.95	3830	−1.43	1.14
WHZ	137	−0.99	1.21	1152	−0.88	1.19	734	−1.09	1.15	3830	−0.96	1.37

**Fig. 2:**
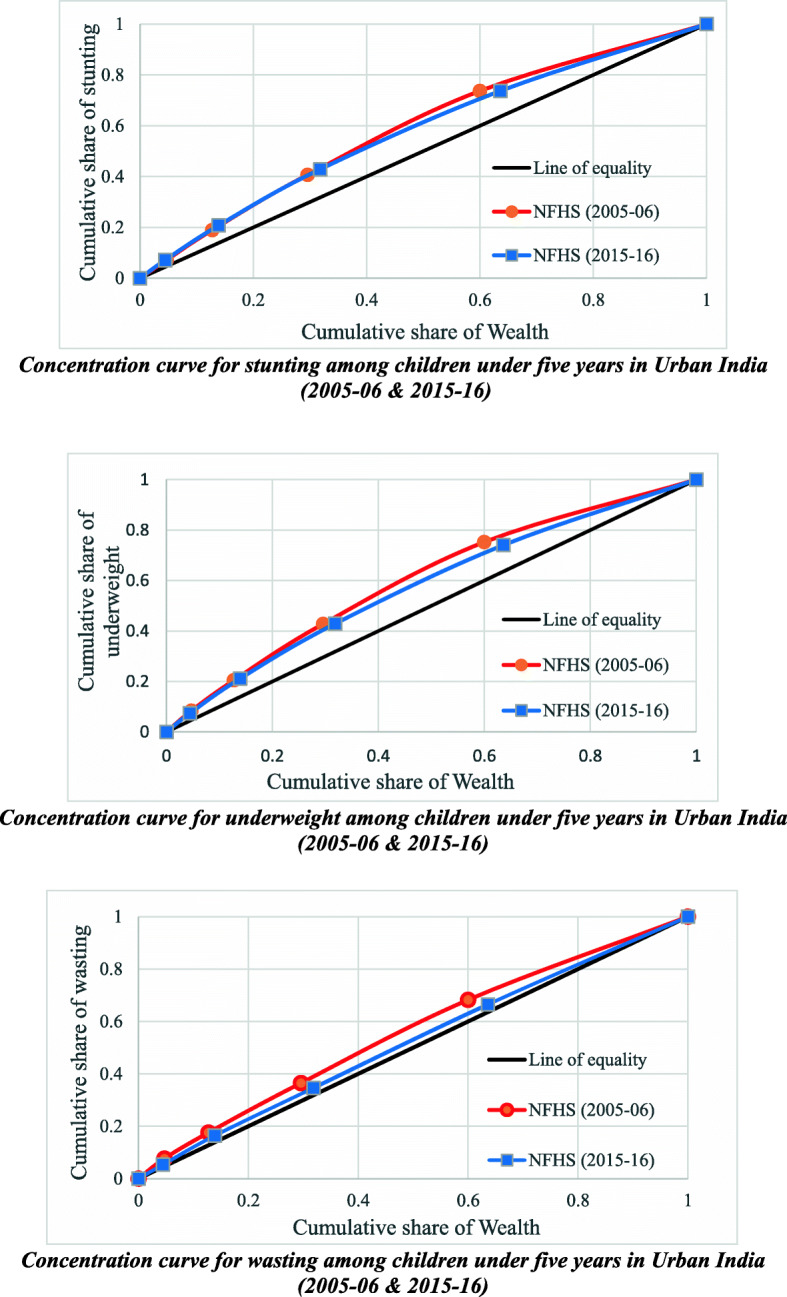
Concentration curves for stunting, underweight and wasting among children under five years in urban India, 2005–06 and 2015–16

Figure 2 depict concentration curves for stunting, wasting, and underweight among children in urban India, during 2005–06 and 2015–16. The result of the concentration curve shows a reduction in the inequality for all three indicators among children below five years in urban India. However, there was a significant decrement in the inequality for stunting, underweight and wasting; the decrement was much faster in wasting (CI: 0.116 [Se: 0.010; *p* < 0.05] in 2005–06 to 0.045 [Se: 0.003; *p* < 0.05] in 2015–16) than in stunting (CI: 0.213 [Se: 0.006; *p* < 0.05] in 2005–06 to 0.162 [Se; 0.004; *p* < 0.05] in 2015–16) or underweight (CI: 0.186 [Se: 0.005; *p* < 0.05] in 2005–06 to 0.156 [Se; 0.004; *p* < 0.05] in 2015–16).

Table 3 presents the estimates of decomposition analysis for the contribution of various explanatory variables for stunting among urban children in India in 2005–06 & 2015–16. The absolute CI for stunting decreased from − 0.186 in 2005–06 to − 0.156 in 2015–16, which depicts that economic inequality has significantly decreased over the two time-periods (0.03; *p* < 0.05). In 2005–06, education of the mother explained about 19% of the observed inequality in the prevalence of stunting among children, which increased to around 27% during 2015–16. Most of the economic inequality in stunting during 2015–16 was explained through mother’s education, parity, defecation, and stool disposal. During 2015–16, education explained 26.6% of the observed inequality in stunting among children, followed by 19% by parity and 14.3% by defecation. During both periods, more than 85% of the economic inequality for stunting among children had been explained by three factors, namely; maternal factors, health-seeking factors, and environmental factors. Maternal factors remained the most significant contributor to inequality during both the time-periods. The contribution of environmental factors in explaining the disparity had increased by about 10% from 13.3% in 2005–06 to 23.2% in 2015–16. However, the contribution of health-seeking factors in explaining the inequality had declined from 30.6% in 2005–06 to 10.4% in 2015–16.

**Table 3:** Estimates of decomposition analysis for contribution of various explanatory variables for stunting among urban children under five years in India, 2005–06 & 2015–16

	NFHS 2005–06				NFHS 2015–16			
Variables	Elasticity	CI	Absolute contribution to CI	Percentage Contribution	Elasticity	CI	Absolute contribution to CI	Percentage Contribution
**Mother factors**								
Age (years)	−0.061	0.019	− 0.001	2.3	− 0.067	0.025	−0.002	5.5
Education	−0.055	0.173	−0.010	19.1	−0.086	0.094	−0.008	26.6
Age at First Birth (years)	−0.110	0.045	−0.005	9.9	0.019	0.023	0.000	−1.5
Parity	0.081	−0.091	− 0.007	14.8	0.076	−0.075	− 0.006	19.0
Anemia	−0.007	−0.076	0.001	−1.1	0.015	−0.051	− 0.001	2.6
**Socio-economic factors**								
Caste	−0.083	0.026	−0.002	4.4	−0.071	0.025	−0.002	5.9
Religion	−0.008	− 0.003	0.000	−0.1	0.003	−0.002	0.000	0.0
**Child factors**								
Age (months)	0.094	− 0.005	0.000	0.9	0.023	0.001	0.000	−0.1
Sex	−0.030	− 0.004	0.000	−0.3	− 0.026	−0.003	0.000	− 0.3
Anemia	0.065	−0.064	−0.004	8.4	0.040	− 0.058	−0.002	7.7
Diarrhea	0.001	−0.025	0.000	0.1	0.000	−0.039	0.000	0.0
Birth weight	0.011	0.098	0.001	−2.2	0.012	− 0.027	0.000	1.1
Colostrum feeding	0.001	0.079	0.000	−0.2	0.002	0.009	0.000	− 0.1
**Environmental factors**								
Source of drinking water	0.035	0.013	0.000	−0.9	0.010	−0.002	0.000	0.1
Defecation	− 0.085	0.066	−0.006	11.4	−0.094	0.046	−0.004	14.3
Stool Disposal	0.007	−0.308	−0.002	4.1	0.009	− 0.308	−0.003	8.9
Regions of India	0.045	0.014	0.001	−1.3	−0.026	−0.002	0.000	−0.2
**Health seeking factors**								
Skilled birth attendant	0.000	0.141	0.000	−0.1	−0.032	0.042	−0.001	4.5
Place of delivery	−0.070	0.163	−0.011	22.8	−0.029	0.050	−0.001	4.9
Full Immunization	−0.016	0.146	−0.002	4.6	0.023	0.046	0.001	−3.5
Full ANC	−0.004	0.360	−0.001	3.2	−0.007	0.208	−0.001	4.5
**Explained CI**			−0.050	100			−0.030	100.00
**Actual CI**			−0.186				−0.156	
**Residual**			−0.136				−0.126	

Table 4 presents the estimates of decomposition analysis for the contribution of various explanatory variables for underweight among urban children in India, during 2005–06 & 2015–16. The actual CI for underweight decreased from − 0.213 in 2005–06 to − 0.162 in 2015–16, which depicts a significant reduction in economic inequality (0.051, *p* < 0.05). In explaining economic inequality for underweight in 2005–06, maternal education (28.8%) was the most significant contributor in explaining the majority of the inequality followed by open defecation (16.9%), and place of delivery (16.7%). In 2015–16, the contribution of maternal education slightly decreased to 25.4%, but remained as a significant contributor in explaining the inequality followed by open defecation (17.5%), and parity (16.3%). In 2015–16, nearly half of the economic disparities in underweight among urban children was explained by maternal factors (49%), followed by environmental factors (25.4%), and health seeking factors (14.4%).

**Table 4:** Estimates of decomposition analysis for contribution of various explanatory variables for underweight among urban children under five years in India, 2005–06 & 2015–16

	NFHS 2005–06				NFHS 2015–16			
Variables	Elasticity	CI	Absolute contribution to CI	Percentage Contribution	Elasticity	CI	Absolute contribution to CI	Percentage Contribution
**Mother factors**								
Age (years)	−0.043	0.019	−0.001	1.8	−0.072	0.025	−0.002	5.96
Education	−0.078	0.173	−0.013	28.8	−0.081	0.094	−0.008	25.44
Age at First Birth (years)	−0.067	0.045	−0.003	6.4	0.004	0.023	0.000	−0.34
Parity	0.041	−0.091	−0.004	7.9	0.064	−0.075	−0.005	16.29
Anemia	0.011	−0.076	−0.001	1.7	0.010	−0.051	−0.001	1.68
**Socio-economic factors**								
Caste	−0.048	0.026	−0.001	2.7	−0.059	0.025	−0.001	5.04
Religion	−0.030	−0.003	0.000	−0.2	− 0.005	−0.002	0.000	0.00
**Child factors**								
Age (months)	0.079	−0.005	0.000	0.8	0.059	0.001	0.000	−0.25
Sex	−0.017	−0.004	0.000	−0.2	− 0.029	−0.003	0.000	−0.25
Anemia	0.033	−0.064	−0.002	4.5	0.025	−0.058	−0.001	4.87
Diarrhea	0.002	−0.025	0.000	0.1	0.001	−0.039	0.000	0.17
Birth weight	0.015	0.098	0.001	−3.2	0.019	−0.027	−0.001	1.68
Colostrum feeding	0.003	0.079	0.000	−0.5	0.002	0.009	0.000	−0.08
**Environmental factors**								
Source of drinking water	0.038	0.013	0.000	−1.0	0.033	−0.002	0.000	0.17
Defecation	−0.119	0.066	−0.008	16.9	−0.112	0.046	−0.005	17.46
Stool Disposal	0.010	−0.308	−0.003	6.7	0.008	−0.308	−0.002	7.81
Regions of India	0.028	0.014	0.000	−0.9	− 0.006	−0.002	0.000	−0.08
**Health seeking factors**								
Skilled birth attendant	−0.001	0.141	0.000	0.3	−0.026	0.042	−0.001	3.61
Place of delivery	−0.048	0.163	−0.008	16.7	−0.045	0.050	−0.002	7.56
Full Immunization	−0.018	0.146	−0.003	5.6	0.002	0.046	0.000	−0.34
Full ANC	−0.006	0.360	−0.002	5.0	−0.005	0.208	−0.001	3.61
**Explained CI**			−0.047	100.00			−0.030	100.00
**Actual CI**			−0.213				−0.162	
**Residual**			−0.166				−0.132	

Table 5 presents the estimates of decomposition analysis for the contribution of various explanatory variables for wasting among urban children in India, 2005–06 & 2015–16. The actual CI for wasting decreased from − 0.116 in 2005–06 to − 0.045 in 2015–16, which depicts that economic inequality has significantly decreased over time (0.071, p < 0.05). Unlike for stunting and underweight, defecation (36.8%) explained the majority of the economic inequality in wasting during 2005–06, which further increased to (46.9%) in 2015–16. The contribution of maternal education in the explanation of economic inequality decreased from (33.4%) in 2005–06 to (11.8%) in 2015–16. In 2015–16, environmental factors (55.9%) explained most of the gap for economic inequality for wasted children in India, followed by maternal factors (22.2%) and Health seeking factors (22.2%).

**Table 5:** Estimates of decomposition analysis for contribution of various explanatory variables for wasting among urban children under five years in India, 2005–06 & 2015–16

	NFHS 2005–06				NFHS 2015–16			
Variables	Elasticity	CI	Absolute contribution to CI	Percentage Contribution	Elasticity	CI	Absolute contribution to CI	Percentage Contribution
**Mother factors**								
Age (Years)	−0.008	0.019	0.000	1.3	−0.004	0.025	0.000	1.4
Education	−0.023	0.173	−0.004	33.4	−0.009	0.094	−0.001	11.8
Age at First Birth (years)	0.006	0.045	0.000	−2.1	−0.004	0.023	0.000	1.4
Parity	0.013	−0.091	−0.001	9.9	0.006	−0.075	0.000	6.6
Anemia	0.003	−0.076	0.000	1.9	0.002	−0.051	0.000	1.0
**Socio-economic factors**								
Caste	−0.034	0.026	−0.001	7.6	−0.009	0.025	0.000	3.1
Religion	−0.004	−0.003	0.000	0.0	−0.010	−0.002	0.000	−0.4
**Child factors**								
Age (months)	−0.068	−0.005	0.000	−2.5	−0.056	0.001	0.000	0.7
Sex	−0.027	−0.004	0.000	−1.1	−0.023	− 0.003	0.000	− 0.7
Anemia	−0.006	− 0.064	0.000	−3.0	− 0.008	−0.058	0.000	−6.6
Diarrhea	0.003	−0.025	0.000	0.4	0.001	−0.039	0.000	0.7
Birth weight	0.007	0.098	0.001	−5.9	0.010	−0.027	0.000	3.5
Colostrum feeding	0.004	0.079	0.000	−2.8	0.004	0.009	0.000	−0.7
**Environmental factors**								
Source of drinking water	0.023	0.013	0.000	−2.3	0.031	−0.002	0.000	0.7
Defecation	−0.066	0.066	−0.004	36.8	−0.072	0.046	−0.003	46.9
Stool Disposal	0.002	−0.308	−0.001	3.8	0.002	−0.308	−0.001	8.0
Regions of India	0.006	0.014	0.000	−0.6	0.017	−0.002	0.000	0.4
**Health seeking factors**								
Skilled birth attendant	0.011	0.141	0.002	−12.7	−0.001	0.042	0.000	0.4
Place of delivery	−0.004	0.163	−0.001	5.3	−0.006	0.050	0.000	3.8
Full Immunization	−0.022	0.146	−0.003	26.4	−0.025	0.046	−0.001	16.0
Full ANC	−0.002	0.360	−0.001	6.1	−0.001	0.208	0.000	2.1
**Explained CI**			−0.012	100.00			−0.007	100.00
**Actual CI**			−0.116				−0.045	
**Residual**			−0.104				−0.038	

## Discussion

Despite recent achievements in economic progress, India failed to ensure better outcomes in child growth. Results further suggested that there exists socio-economic clustering that is further widening the gap in securing development among children. Through this study, we have tried to explain the factors contributing to economic inequality in stunting, underweight, and wasting among children below five years in urban India.

There is little data to understand the timing of growth faltering in under-five age children in India, and whatever limited study conducted to understand the growth faltering among children measured growth faltering through reference growth standards of NCHS (National Centre for Health Statistics) methodology [25]. This study has the edge over previous studies in measuring growth faltering as this study used WHO standards to measure the growth faltering among children over NCHS methodology [34]. The result confirms that wasting among urban poor and non-poor children was more prone during the first year of birth, and stunting among urban poor and non-poor children is more prone during the second year of birth. Growth flattering in underweight is more during the second year of birth among urban poor children and the fifth year of birth among urban non-poor children. Critically, children aged 21–25 months were more prone to growth faltering than the children of any other age group. Studies found that children in low-middle-income countries experience rapid growth faltering during the first two years of life [40].

Maternal factors remained a prime contributor to inequality. Stunting in simple terms is defined as - > 2 SD height-for-age [38], and measures the past or chronic child undernutrition. The mother’s related factors explained a little more than half of the inequality for stunting among children during 2015–16. Various studies in Indian set up are in coordination with this study, concluded that maternal factors such as mother’s age mother’s education, age at first birth, parity, and anaemia were highly associated with stunting [28, 36]. Of all the mother’s factors, mother’s education is the most significant contributor to the economic inequality in defining stunting among children. Not only in India [10], but in developed [44] and developing countries [2, 20] also, mother’s education was found significantly associated with stunting among children. Among environmental factors, defecation and stool disposal explained the highest economic inequality in stunting among children. A study conducted in Rwanda for 10 years, using decomposition analysis reported that defecation has no association with stunting [30]. Another study conducted in rural Zimbabwe employing cluster-randomized trials found that WASH (Water, Sanitation, and Hygiene) practices implemented in rural areas are unlikely to reduce stunting [16]. However, few studies found that sanitation and defecation significantly affect stunting among children [24]. A study conducted in Indian settings utilizing NFHS data concluded that the practice of open defecation influences the inequality in stunting [35]. Health seeking factors explained about 30% of the inequality in stunting in 2005–06, and in 2015–16, it could only explain about 10% of the inequality. From various works of literature, it can be summed up that full Antenatal Care and Skill attendants at birth had increased over time in India [1].

Underweight is defined as - > 2 SD weight-for-age, and it depicts the past as well as present undernutrition. During both the time, mother’s factors remained the highest contributory factors that explained the inequality in underweight among children aged 0–5 years. Employing the decomposition approach, a study concluded that maternal factors improve WAZ among children aged 0–5 years in India [27]. In other studies also, the role of mother’s education in explaining the inequalities in underweight was highlighted in Indian settings [29, 35]. Of the environmental factors, defecation remained the highest contributor to the inequality in underweight with nearly 17%, during both the time-periods. Open defecation is a problem among masses in India, and recently the Indian Government has done well to tackle the issue [6]. On the base of the decomposition approach, a study conducted in India confirmed that open defecation positively influences the inequality in underweight [35]. A study conducted in five Indian states; Uttar Pradesh, Karnataka, Madhya Pradesh, Rajasthan, and West Bengal, found that children from open defecation free (ODF) districts were less underweight than children in non-ODF districts [37], thus confirming the importance of open defecation in understanding the inequality in underweight. The result found that health-seeking factors explained nearly one-fourth (27.7%) of the inequality in underweight in 2005–06, but the same declined to nearly 14.4% in 2015–16. The decline in the contribution of the place of delivery in explaining the inequality signifies the equitable access of institutional deliveries in urban areas among poor and non-poor. A study found that in India, the utilization of institutional delivery care has increased from 43% in 2004 to 83% in 2014 [21]. This increase in institutional delivery had helped in shrinking the gap of inequality in underweight, as explained by place of delivery.

Wasting is defined as - > 2 SD weight-for-height among children. It is a measure of current nutrition status, also known as acute undernutrition. The inequality in wasting among children has decreased, but the prevalence of wasting has increased. Around the world, wasting had reduced at a plodding pace over the last 40 years, but in countries like India and Sri Lanka, wasting has increased [39]. Maternal factors (44.4%) were the highest contributory factors in explaining the inequality in wasting among children during 2005–06, but the contribution of maternal factors in inequality in wasting had declined significantly to 22.2% in 2015–16. In previous studies, maternal factors were deemed as the contributory factors in explaining the inequalities in wasting among children aged below five years [23]. Nearly half of the inequality in wasting among children had been explained by open defecation (46.9%) in 2015–16, citing the importance of sanitation. Since the weight-for-height is a measure of current nutritional status and is likely to be affected by small illnesses episodes and infectious diseases [23], the contribution of defecation in inequality in wasting among children is highly visible. Unavailability of toilet facilities within households promotes open defecation, and in turn, it facilitates water-borne diseases, which negatively affect the current health and nutrition status among children. Open defecation reinforces under-nutrition (wasting) via increased susceptibility to infections and through reducing immunity [31].

### Strengths and limitations

One of the strengths of the study is the considerable sample size for both the survey periods. This study explores the inequalities in stunting, underweight, and stunting among urban poor by employing survey data, which is 10 years apart, thus giving a clear picture of decadal change in economic inequality in stunting, underweight, and wasting among urban children. Decomposition allows our understanding of various factors contributing to the disparities inequality in stunting, underweight, and wasting among urban children. This study segregated the covariates of economic inequality into five categories; mother’s factor, health-seeking factors, environmental factors, child factors, and socio-economic factors, thus highlighting the importance of particular covariate in explaining the inequality. The cross-sectional nature of data limits our knowledge about the causation accurately. Maternal factors used in this article may not be exclusively maternal physiological or maternal factors as such only, some of them are more of socio-economic nature (e.g. education). Educational status is commonly used as a proxy of socio-economic status.

## Conclusions

The study made a reasonable attempt to describe the growth pattern among children below five along with the inequality in stunting, underweight, and wasting among children in urban India. Several important observations emerge from this study. First, during 2005–06 as well as 2015–16, maternal factors (specifically mother’s education) were the highest contributory factors in explaining rich-poor inequality in stunting and underweight among urban children. Second, maternal factors were the most significant contributory factors in explaining the wasting among children during 2005–06, however environmental factors (specifically open defecation) became the most significant contributory factors in explaining the rich-poor inequality in wasting among children during 2015–16. Third, more than 85% of the inequality in stunting, underweight, and wasting among urban children was explained by maternal factors, environmental factors, and health-seeking factors. Fourth, growth faltering among children reaches its peak by the end of 2 years of age.

Rich-poor inequality in malnutrition calls out for the expedite policy interventions aimed at poverty reduction in urban areas. There is also a need to provide mass education regarding nutrition and health, along with focussing on the correlates that aim at improving mother’s education. Environmental factors also promoted the inequality in stunting, wasting, and underweight in urban children; there is a need to focus the enigma of open defecation in India. After the launch of Swachh Bharat Mission in 2014, the government of India has focused a lot on building individual household latrines [6]. Despite having access to latrines, people prefer to defecate in open [3, 8, 18, 32]. There is a need to promote the use of latrines among Indian masses. There is a need to bring a change in behavioural aspects and preferences of people to promote latrine use. Information, Education, and Communication (IEC) activities are encouraged to clamp down the economic inequality in stunting, underweight, and wasting among urban children. Since the cognitive and developmental deficiency resulting from malnutrition may significantly be irreversible after two years of age [7], it is being understood that the ‘window of opportunity’ for preventing malnutrition ends at age two years. It is therefore encouraged that policy-makers shall evenly target children for nutritional programs, with emphasis on children aged below two years.

All the nutrition-specific and nutrition-sensitive interventions in urban areas should be prioritized, focusing on urban poor, who are often clustered in low-income slums. Rich-poor inequality in child growth faltering calls out for integration and convergence of nutrition interventions with policy intervention aimed at poverty reduction in urban areas. There is also a need to expand the scope of the ICDS program to provide mass education regarding nutrition and health by making provisions of home visits primarily focusing at pregnant and lactating mothers.

## Supplementary Information

**Additional file 1:** **Figure-S1.** Poor and non-poor differentials for growth patterns among children in urban India, 2005–06 and 2015–16**. Table- S1** Mean and standard error estimates for HAZ, WAZ and WHZ scores among children under 5 years in urban India, 2005–06. **Table- S2** Mean and standard error estimates for HAZ, WAZ and WHZ scores among children under 5 years in urban India, 2015–16

## Data Availability

The study utilises secondary source of data which is freely available in public domain through http://iipsindia.org.

## Abbreviations

ANC: Ante Natal Care
BCG: Bacillus Calmette-Guerin
CC: Concentration Curve
CI: Concentration Index
DPT: Diphtheria, Pertussis, and Tetanus
GHI: Global Hunger Index
HAZ: Height-for-Age Z score
ICDS: Integrated Child Development Services
IEC: Information, Education, and Communication
IFA: Iron Folic Acid
IIPS: International Institute for Population Sciences
NCHS: National Centre for Health Statistics
NFHS: National Family Health Survey
ODF: Open Defecation Free
SC: Schedule Caste
ST: Schedule Tribe
UNICEF: United Nations International Children’s Emergency Fund
WAZ: Weight-for-Age Z score
WHO: World Health Organization
WHZ: Weight-for-Height Z score
Se: Standard error
SD: Standard deviation
