# Supplementary material for: Inequality in child undernutrition among urban population in India: a decomposition analysis
Source: BMC Public Health. 2020 Dec 3;20:1852. doi: 10.1186/s12889-020-09864-2 (PMC7713021; doi:10.1186/s12889-020-09864-2)
Supplement: Supplementary file 1 — Additional file 1: Figure-S1. Poor and non-poor differentials for growth patterns among children in urban India, 2005–06 and 2015–16. Table- S1 Mean and standard error estimates for HAZ, WAZ and WHZ scores among children under 5 years in urban India, 2005–06. Table- S2 Mean and standard error estimates for HAZ, WAZ and WHZ scores among children under 5 years in urban India, 2015–16 [file 12889_2020_9864_MOESM1_ESM.docx]

| **Figure-S1** Poor and non-poor differentials for growth patterns among children in urban India, 2005-06 and 2015-16 | |
| --- | --- |
| **Height for Age (HAZ)** | |
|  |  |
| **Weight for Age (WAZ)** | |
|  |  |
| **Weight for Height (WHZ)** | |
|  |  |

| **Table- S1 Mean and standard error estimates for HAZ, WAZ and WHZ scores among children under five years in urban India, 2005-06** | | | | | | |
| --- | --- | --- | --- | --- | --- | --- |
|  | **HAZ (Height for age)** | | **WAZ (Weight for age)** | | **WHZ (Weight for height)** | |
| **Age (months)** | **Non-Poor** | **Poor** | **Non-Poor** | **Poor** | **Non-Poor** | **Poor** |
|  | **(Mean, St. Error)** | **(Mean, St. Error)** | **(Mean, St. Error)** | **(Mean, St. Error)** | **(Mean, St. Error)** | **(Mean, St. Error)** |
| 0 | (-0.37,0.23) | (-0.46,0.45) | (-0.46,0.21) | (-0.48,0.44) | (-0.62,0.3) | (-0.57,0.54) |
| 1 | (-0.72,0.17) | (-1.29,0.34) | (-1.03,0.13) | (-1.83,0.34) | (-0.75,0.19) | (-1.07,0.3) |
| 2 | (-0.4,0.14) | (-0.57,0.32) | (-1.07,0.1) | (-1.23,0.36) | (-0.92,0.13) | (-1.03,0.41) |
| 3 | (-0.03,0.15) | (-0.88,0.44) | (-1.19,0.09) | (-2.02,0.33) | (-1.47,0.15) | (-1.87,0.31) |
| 4 | (-0.39,0.12) | (-1.6,0.4) | (-1.06,0.08) | (-1.79,0.29) | (-0.91,0.12) | (-0.64,0.32) |
| 5 | (-0.6,0.11) | (-1.73,0.48) | (-1.27,0.09) | (-2.04,0.31) | (-1.08,0.1) | (-0.91,0.41) |
| 6 | (-0.45,0.11) | (-1.38,0.32) | (-1.09,0.08) | (-2.33,0.24) | (-0.95,0.11) | (-1.83,0.2) |
| 7 | (-0.56,0.11) | (-1.56,0.41) | (-0.99,0.08) | (-2.05,0.33) | (-0.75,0.11) | (-1.33,0.35) |
| 8 | (-0.73,0.12) | (-1.58,0.31) | (-0.93,0.09) | (-1.8,0.31) | (-0.55,0.11) | (-1.12,0.33) |
| 9 | (-0.7,0.1) | (-0.83,0.36) | (-1.12,0.08) | (-1.87,0.23) | (-0.91,0.09) | (-1.78,0.3) |
| 10 | (-0.76,0.1) | (-0.81,0.21) | (-1.1,0.07) | (-1.2,0.22) | (-0.9,0.09) | (-1.03,0.29) |
| 11 | (-0.96,0.11) | (-2.16,0.39) | (-1.05,0.09) | (-1.83,0.31) | (-0.72,0.1) | (-0.86,0.66) |
| 12 | (-1.39,0.1) | (-1.76,0.18) | (-1.36,0.08) | (-1.91,0.19) | (-0.88,0.1) | (-1.42,0.24) |
| 13 | (-1.15,0.13) | (-2.18,0.36) | (-1.25,0.08) | (-2.17,0.31) | (-0.92,0.09) | (-1.41,0.2) |
| 14 | (-1.4,0.1) | (-2.03,0.33) | (-1.19,0.08) | (-2.15,0.26) | (-0.7,0.09) | (-1.56,0.2) |
| 15 | (-1.75,0.11) | (-2.4,0.21) | (-1.45,0.08) | (-2,0.17) | (-0.81,0.09) | (-1.13,0.24) |
| 16 | (-1.45,0.1) | (-2.01,0.42) | (-1.09,0.08) | (-2.31,0.25) | (-0.54,0.08) | (-1.8,0.26) |
| 17 | (-1.7,0.09) | (-1.56,0.39) | (-1.45,0.07) | (-1.6,0.21) | (-0.87,0.08) | (-1.16,0.22) |
| 18 | (-1.73,0.09) | (-2.61,0.26) | (-1.31,0.07) | (-2.11,0.28) | (-0.65,0.07) | (-1.18,0.29) |
| 19 | (-1.43,0.13) | (-2.5,0.27) | (-1.06,0.11) | (-2.19,0.22) | (-0.52,0.11) | (-1.32,0.28) |
| 20 | (-1.5,0.1) | (-3,0.35) | (-1.44,0.07) | (-2.1,0.27) | (-0.98,0.08) | (-0.84,0.27) |
| 21 | (-1.86,0.1) | (-2.19,0.3) | (-1.51,0.08) | (-1.97,0.24) | (-0.78,0.08) | (-1.2,0.25) |
| 22 | (-1.96,0.09) | (-2.89,0.23) | (-1.54,0.08) | (-2.03,0.21) | (-0.76,0.09) | (-0.75,0.24) |
| 23 | (-1.77,0.11) | (-3.22,0.39) | (-1.43,0.07) | (-2.9,0.63) | (-0.72,0.08) | (-1.86,0.72) |
| 24 | (-1.74,0.11) | (-2.4,0.41) | (-1.45,0.09) | (-2.14,0.24) | (-0.77,0.08) | (-1.21,0.16) |
| 25 | (-1.46,0.1) | (-2.66,0.26) | (-1.35,0.07) | (-2.2,0.19) | (-0.82,0.07) | (-1.09,0.15) |
| 26 | (-1.62,0.09) | (-1.41,0.53) | (-1.32,0.07) | (-1.76,0.26) | (-0.65,0.08) | (-1.42,0.34) |
| 27 | (-1.65,0.1) | (-2.76,0.27) | (-1.44,0.07) | (-2.25,0.23) | (-0.8,0.08) | (-1.05,0.22) |
| 28 | (-1.74,0.09) | (-2.56,0.27) | (-1.5,0.08) | (-2,0.2) | (-0.77,0.07) | (-0.89,0.22) |
| 29 | (-1.59,0.1) | (-2.06,0.21) | (-1.3,0.08) | (-1.96,0.17) | (-0.63,0.08) | (-1.15,0.14) |
| 30 | (-1.57,0.09) | (-2.56,0.38) | (-1.33,0.06) | (-2.06,0.2) | (-0.66,0.08) | (-0.86,0.29) |
| 31 | (-1.71,0.09) | (-1.99,0.38) | (-1.47,0.07) | (-1.86,0.3) | (-0.76,0.07) | (-1.09,0.21) |
| 32 | (-1.6,0.12) | (-3.03,0.26) | (-1.39,0.09) | (-2.66,0.21) | (-0.71,0.09) | (-1.38,0.23) |
| 33 | (-1.82,0.12) | (-2.74,0.39) | (-1.7,0.08) | (-2.46,0.23) | (-0.99,0.09) | (-1.29,0.24) |
| 34 | (-1.92,0.11) | (-2.7,0.28) | (-1.58,0.08) | (-2.1,0.19) | (-0.71,0.08) | (-0.8,0.18) |
| 35 | (-2.02,0.12) | (-3.45,0.3) | (-1.8,0.09) | (-2.92,0.21) | (-0.95,0.08) | (-1.36,0.22) |
| 36 | (-1.97,0.12) | (-1.74,0.35) | (-1.69,0.09) | (-1.67,0.23) | (-0.83,0.08) | (-0.95,0.22) |
| 37 | (-1.48,0.09) | (-2.5,0.24) | (-1.45,0.07) | (-2.07,0.17) | (-0.89,0.08) | (-0.93,0.14) |
| 38 | (-1.55,0.11) | (-2.61,0.33) | (-1.48,0.08) | (-2.11,0.15) | (-0.87,0.07) | (-0.84,0.21) |
| 39 | (-1.43,0.08) | (-2.24,0.29) | (-1.36,0.07) | (-1.95,0.17) | (-0.8,0.08) | (-0.94,0.22) |
| 40 | (-1.61,0.1) | (-2.6,0.32) | (-1.32,0.08) | (-1.79,0.21) | (-0.58,0.08) | (-0.38,0.27) |
| 41 | (-1.73,0.08) | (-2.3,0.49) | (-1.61,0.06) | (-1.96,0.5) | (-0.89,0.07) | (-0.87,0.41) |
| 42 | (-1.55,0.1) | (-3.02,0.24) | (-1.44,0.07) | (-2.59,0.21) | (-0.79,0.07) | (-1.2,0.2) |
| 43 | (-1.78,0.1) | (-2.18,0.32) | (-1.61,0.08) | (-1.77,0.21) | (-0.85,0.09) | (-0.74,0.33) |
| 44 | (-1.84,0.09) | (-2.27,0.21) | (-1.53,0.07) | (-2,0.21) | (-0.65,0.07) | (-0.96,0.24) |
| 45 | (-1.66,0.1) | (-3.1,0.26) | (-1.46,0.07) | (-2.28,0.21) | (-0.7,0.07) | (-0.68,0.22) |
| 46 | (-2.01,0.11) | (-2.43,0.26) | (-1.87,0.08) | (-2.37,0.19) | (-1,0.08) | (-1.35,0.18) |
| 47 | (-2.13,0.12) | (-2.99,0.29) | (-1.81,0.09) | (-2.51,0.28) | (-0.78,0.09) | (-1.07,0.27) |
| 48 | (-1.9,0.1) | (-2.89,0.39) | (-1.71,0.07) | (-2.59,0.24) | (-0.87,0.08) | (-1.19,0.46) |
| 49 | (-1.53,0.11) | (-1.47,0.35) | (-1.56,0.08) | (-1.04,0.36) | (-0.95,0.09) | (-0.22,0.31) |
| 50 | (-1.38,0.1) | (-2.64,0.22) | (-1.34,0.08) | (-2.44,0.21) | (-0.76,0.08) | (-1.23,0.2) |
| 51 | (-1.31,0.09) | (-2.3,0.2) | (-1.41,0.08) | (-1.8,0.2) | (-0.93,0.08) | (-0.62,0.22) |
| 52 | (-1.49,0.08) | (-2.96,0.21) | (-1.28,0.06) | (-2.31,0.15) | (-0.56,0.08) | (-0.76,0.2) |
| 53 | (-1.64,0.08) | (-2.4,0.22) | (-1.57,0.07) | (-2.18,0.21) | (-0.87,0.07) | (-1.1,0.25) |
| 54 | (-1.46,0.09) | (-2.82,0.23) | (-1.4,0.08) | (-2.5,0.18) | (-0.79,0.09) | (-1.2,0.24) |
| 55 | (-1.66,0.09) | (-2.41,0.22) | (-1.55,0.08) | (-2.06,0.24) | (-0.84,0.08) | (-0.91,0.32) |
| 56 | (-1.58,0.09) | (-2.74,0.33) | (-1.53,0.07) | (-2.14,0.31) | (-0.88,0.07) | (-0.76,0.33) |
| 57 | (-1.6,0.09) | (-2.17,0.25) | (-1.56,0.08) | (-1.97,0.15) | (-0.89,0.07) | (-0.98,0.15) |
| 58 | (-1.35,0.08) | (-2.45,0.18) | (-1.34,0.08) | (-2.14,0.15) | (-0.81,0.08) | (-0.99,0.21) |
| 59 | (-1.78,0.09) | (-2.85,0.24) | (-1.73,0.07) | (-2.59,0.13) | (-1,0.08) | (-1.28,0.16) |

| **Table- S2 Mean and standard error estimates for HAZ, WAZ and WHZ scores among children under five years in urban India, 2015-16** | | | | | | |
| --- | --- | --- | --- | --- | --- | --- |
|  | **HAZ (Height for age)** | | **WAZ (Weight for age)** | | **WHZ (Weight for height)** | |
| **Age (months)** | **Non-Poor** | **Poor** | **Non-Poor** | **Poor** | **Non-Poor** | **Poor** |
|  | **(Mean, St. Error)** | **(Mean, St. Error)** | **(Mean, St. Error)** | **(Mean, St. Error)** | **(Mean, St. Error)** | **(Mean, St. Error)** |
| 0 | (-0.26,0.11) | (-0.91,0.26) | (-0.98,0.07) | (-1.25,0.19) | (-1.57,0.13) | (-1.18,0.23) |
| 1 | (-0.49,0.08) | (-0.54,0.15) | (-1.15,0.05) | (-0.92,0.11) | (-1.24,0.08) | (-0.83,0.18) |
| 2 | (-0.27,0.08) | (-0.69,0.19) | (-1.17,0.06) | (-1.72,0.14) | (-1.2,0.08) | (-1.54,0.17) |
| 3 | (-0.33,0.07) | (-1.02,0.17) | (-1.25,0.05) | (-1.61,0.11) | (-1.26,0.07) | (-0.95,0.15) |
| 4 | (-0.28,0.07) | (-1.05,0.18) | (-1.2,0.05) | (-1.63,0.11) | (-1.23,0.07) | (-0.97,0.16) |
| 5 | (-0.28,0.07) | (-0.98,0.16) | (-1.09,0.04) | (-1.7,0.12) | (-1.09,0.06) | (-1.26,0.15) |
| 6 | (-0.34,0.07) | (0.01,0.18) | (-1.13,0.04) | (-1.4,0.11) | (-1.07,0.06) | (-1.67,0.11) |
| 7 | (-0.19,0.07) | (-0.94,0.17) | (-0.95,0.05) | (-1.61,0.1) | (-0.96,0.06) | (-1.22,0.13) |
| 8 | (-0.34,0.07) | (-0.83,0.18) | (-1.05,0.05) | (-1.42,0.13) | (-1.02,0.06) | (-1.09,0.13) |
| 9 | (-0.52,0.07) | (-1.03,0.14) | (-1.1,0.05) | (-1.72,0.11) | (-0.98,0.06) | (-1.46,0.13) |
| 10 | (-0.56,0.08) | (-1.35,0.18) | (-1.08,0.05) | (-1.7,0.14) | (-0.96,0.06) | (-1.24,0.15) |
| 11 | (-0.92,0.07) | (-1.31,0.16) | (-1.16,0.05) | (-1.68,0.1) | (-0.86,0.06) | (-1.3,0.13) |
| 12 | (-0.91,0.07) | (-1.46,0.16) | (-1.07,0.04) | (-1.44,0.13) | (-0.79,0.05) | (-0.9,0.14) |
| 13 | (-0.92,0.07) | (-1.35,0.18) | (-1.08,0.05) | (-1.72,0.1) | (-0.81,0.05) | (-1.39,0.12) |
| 14 | (-1.1,0.07) | (-2.08,0.15) | (-1.11,0.05) | (-1.98,0.1) | (-0.78,0.06) | (-1.29,0.12) |
| 15 | (-1.15,0.07) | (-1.49,0.17) | (-1.16,0.05) | (-1.65,0.1) | (-0.82,0.06) | (-1.28,0.12) |
| 16 | (-1.28,0.06) | (-2.12,0.14) | (-1.15,0.04) | (-1.88,0.11) | (-0.74,0.05) | (-1.18,0.13) |
| 17 | (-1.28,0.06) | (-1.78,0.18) | (-1.16,0.04) | (-1.66,0.13) | (-0.77,0.05) | (-1.06,0.11) |
| 18 | (-1.23,0.06) | (-1.77,0.14) | (-1.18,0.04) | (-1.83,0.1) | (-0.81,0.05) | (-1.35,0.1) |
| 19 | (-1.39,0.06) | (-1.63,0.13) | (-1.24,0.04) | (-1.74,0.09) | (-0.78,0.05) | (-1.31,0.09) |
| 20 | (-1.39,0.06) | (-2.38,0.11) | (-1.23,0.04) | (-2.08,0.1) | (-0.77,0.05) | (-1.23,0.11) |
| 21 | (-1.53,0.06) | (-1.62,0.16) | (-1.23,0.05) | (-1.74,0.09) | (-0.67,0.05) | (-1.3,0.09) |
| 22 | (-1.37,0.07) | (-2.39,0.15) | (-1.41,0.05) | (-2.08,0.1) | (-1,0.05) | (-1.2,0.1) |
| 23 | (-1.6,0.06) | (-2.16,0.14) | (-1.27,0.05) | (-2.06,0.09) | (-0.64,0.05) | (-1.32,0.11) |
| 24 | (-1.22,0.07) | (-2.22,0.14) | (-1.28,0.05) | (-2.09,0.11) | (-0.91,0.05) | (-1.32,0.11) |
| 25 | (-0.72,0.08) | (-1.65,0.16) | (-1.05,0.05) | (-1.82,0.11) | (-0.96,0.05) | (-1.35,0.11) |
| 26 | (-1.04,0.06) | (-2.3,0.13) | (-1.16,0.04) | (-2.18,0.11) | (-0.87,0.05) | (-1.35,0.11) |
| 27 | (-1.13,0.06) | (-1.85,0.16) | (-1.26,0.04) | (-1.63,0.1) | (-0.94,0.04) | (-0.88,0.11) |
| 28 | (-1.2,0.06) | (-2.02,0.14) | (-1.38,0.04) | (-1.81,0.09) | (-1.04,0.05) | (-0.99,0.1) |
| 29 | (-1.25,0.06) | (-1.95,0.13) | (-1.31,0.04) | (-2.06,0.1) | (-0.91,0.04) | (-1.43,0.12) |
| 30 | (-1.49,0.06) | (-1.99,0.13) | (-1.38,0.04) | (-1.84,0.11) | (-0.82,0.05) | (-1.04,0.11) |
| 31 | (-1.19,0.06) | (-1.89,0.13) | (-1.25,0.04) | (-1.67,0.09) | (-0.86,0.05) | (-0.87,0.08) |
| 32 | (-1.39,0.05) | (-2.07,0.12) | (-1.33,0.04) | (-2.02,0.09) | (-0.81,0.05) | (-1.24,0.11) |
| 33 | (-1.36,0.05) | (-1.66,0.11) | (-1.35,0.04) | (-1.71,0.08) | (-0.88,0.05) | (-1.12,0.08) |
| 34 | (-1.45,0.05) | (-2.01,0.13) | (-1.4,0.04) | (-1.88,0.09) | (-0.86,0.05) | (-1.07,0.12) |
| 35 | (-1.2,0.06) | (-2.25,0.13) | (-1.34,0.05) | (-2,0.1) | (-0.96,0.05) | (-1.04,0.1) |
| 36 | (-1.31,0.06) | (-2.17,0.11) | (-1.33,0.04) | (-2,0.07) | (-0.86,0.05) | (-1.09,0.09) |
| 37 | (-1.27,0.05) | (-1.86,0.15) | (-1.36,0.04) | (-1.77,0.1) | (-0.95,0.05) | (-1.03,0.11) |
| 38 | (-1.3,0.05) | (-1.74,0.12) | (-1.43,0.04) | (-1.98,0.09) | (-1.02,0.05) | (-1.47,0.12) |
| 39 | (-1.31,0.05) | (-1.87,0.12) | (-1.4,0.04) | (-1.74,0.08) | (-0.96,0.05) | (-0.96,0.1) |
| 40 | (-1.13,0.05) | (-1.96,0.11) | (-1.19,0.04) | (-1.95,0.1) | (-0.8,0.05) | (-1.18,0.13) |
| 41 | (-1.2,0.05) | (-2.14,0.12) | (-1.22,0.04) | (-2.08,0.08) | (-0.78,0.04) | (-1.2,0.1) |
| 42 | (-1.25,0.05) | (-1.66,0.14) | (-1.33,0.04) | (-1.68,0.09) | (-0.9,0.04) | (-1.07,0.1) |
| 43 | (-1.2,0.05) | (-2.14,0.12) | (-1.35,0.04) | (-1.94,0.09) | (-0.98,0.05) | (-1,0.1) |
| 44 | (-1.34,0.05) | (-1.7,0.13) | (-1.32,0.05) | (-1.82,0.09) | (-0.8,0.05) | (-1.22,0.09) |
| 45 | (-1.2,0.05) | (-2.27,0.09) | (-1.36,0.04) | (-2.22,0.08) | (-0.98,0.05) | (-1.31,0.08) |
| 46 | (-1.38,0.05) | (-2.06,0.1) | (-1.45,0.04) | (-2.02,0.09) | (-0.95,0.05) | (-1.2,0.1) |
| 47 | (-1.39,0.05) | (-1.76,0.13) | (-1.44,0.04) | (-1.89,0.09) | (-0.93,0.05) | (-1.25,0.1) |
| 48 | (-1.26,0.05) | (-1.98,0.11) | (-1.37,0.04) | (-2.07,0.07) | (-0.93,0.04) | (-1.32,0.09) |
| 49 | (-1.3,0.06) | (-1.56,0.14) | (-1.31,0.04) | (-1.63,0.11) | (-0.81,0.05) | (-1.06,0.12) |
| 50 | (-1.28,0.05) | (-2.03,0.11) | (-1.31,0.04) | (-1.81,0.09) | (-0.82,0.05) | (-0.89,0.1) |
| 51 | (-1.28,0.05) | (-1.91,0.12) | (-1.37,0.04) | (-1.95,0.09) | (-0.91,0.05) | (-1.22,0.12) |
| 52 | (-1.43,0.04) | (-1.83,0.11) | (-1.41,0.04) | (-1.75,0.09) | (-0.83,0.05) | (-0.96,0.08) |
| 53 | (-1.16,0.05) | (-1.99,0.11) | (-1.27,0.05) | (-1.92,0.08) | (-0.89,0.05) | (-1.07,0.08) |
| 54 | (-1.29,0.05) | (-1.78,0.11) | (-1.42,0.04) | (-1.73,0.11) | (-0.99,0.05) | (-1,0.11) |
| 55 | (-1.21,0.05) | (-1.93,0.1) | (-1.3,0.04) | (-1.89,0.07) | (-0.87,0.05) | (-1.09,0.08) |
| 56 | (-1.19,0.04) | (-2.19,0.09) | (-1.4,0.04) | (-2.04,0.07) | (-1.05,0.05) | (-1.07,0.09) |
| 57 | (-1.43,0.04) | (-2.1,0.12) | (-1.46,0.04) | (-1.95,0.1) | (-0.91,0.05) | (-1.03,0.1) |
| 58 | (-1.4,0.04) | (-2.06,0.1) | (-1.44,0.04) | (-2.1,0.07) | (-0.92,0.05) | (-1.33,0.09) |
| 59 | (-1.47,0.05) | (-2.16,0.09) | (-1.54,0.04) | (-1.94,0.08) | (-1.02,0.05) | (-0.97,0.1) |
